# Epidermal growth factor receptor inhibitors as adjuvant treatment for patients with resected non-small cell lung cancer harboring EGFR mutation: a meta-analysis of randomized controlled clinical trials

**DOI:** 10.1186/s12957-023-02925-x

**Published:** 2023-02-13

**Authors:** Ning Zhao, Zhuo-peng Wu, Jie Yang, Wei-neng Feng, Sheng-li Yang, Ying Luo, Jun Ye, Fei Wang, Xiao-wen Zhang, Ye Xiao, Ling-ling Wu, Wei-quan Gu

**Affiliations:** 1 Department of Thoracic Surgery, The First People’s Hospital of Foshan, Foshan, 528000 Guangdong China; 2 Department of Head and Neck/Thoracic Medical Oncology, The First People’s Hospital of Foshan, Foshan, China; 3 Department of Traditional Chinese Medicine, The First People’s Hospital of Foshan, Foshan, China

**Keywords:** Adjuvant treatment, Non-small cell lung cancer, EGFR-TKI, EGFR Mutation, Meta-analysis

## Abstract

**Background:**

The epidermal growth factor receptor (EGFR) tyrosine kinase inhibitors (TKIs) is still under investigation as adjuvant treatment for early-stage disease. Here, we performed a meta-analysis to evaluate the efficacy of adjuvant EGFR-TKI versus non-EGFR-TKI treatment in patients with completely resected non-small cell lung cancer (NSCLC) harboring EGFR mutation.

**Methods:**

Two investigators independently extracted data from databases. A meta-analysis was performed following the guidelines of the Preferred Reporting Items for Systematic Reviews and Meta-Analyses (PRISMA) statement. The protocol was registered in PROSPERO (ID: CRD42022316481). The primary outcome was disease-free survival (DFS) in patients with EGFR mutation, measured as the hazard ratio (HR). Other outcomes (of subgroup analyses) included overall survival (OS) and DFS.

**Results:**

After the systematic screening, eight studies with a total of 3098 patients with stage IB–IIIA NSCLC were included. The results show that in patients with EGFR mutation, the DFS in the adjuvant EGFR-TKI group was significantly superior to that in the control group, with a HR of 0.47 (95% confidence interval [CI]: 0.30–0.74; *P* = 0.001). In subgroup analyses of DFS, the benefit was observed in the EGFR-TKI group versus the chemotherapy group (HR 0.50, 95% CI 0.30–0.84; *P* = 0.009), the EGFR-TKI combined with chemotherapy group versus the chemotherapy group (HR 0.37, 95% CI 0.16–0.85; *P* = 0.02), and in stage IIA–IIIA NSCLC (HR 0.45, 95% CI 0.27–0.74; *P* = 0.002). However, the benefit of DFS did not translate into improved OS in the whole population (HR 0.79, 95% CI 0.54–1.14; *P* = 0.20).

**Conclusion:**

EGFR-TKIs prolonged DFS but not OS in patients with completely resected stage II–IIIA NSCLC harboring EGFR mutation. Longer follow-ups and new clinical trials that can result in changes in clinical practice are needed.

**Supplementary Information:**

The online version contains supplementary material available at 10.1186/s12957-023-02925-x.

## Introduction

Lung cancer is a leading cause of cancer-related deaths worldwide. Only 20–25% of patients with non-small cell lung cancer (NSCLC) receive surgery [1]. Postoperative cisplatin-based adjuvant chemotherapy is recommended for patients with completely resected stage II–IIIA disease [2]. Pooled analysis by the Lung Adjuvant Cisplatin Evaluation (LACE) Collaborative Group showed that cisplatin-based adjuvant chemotherapy significantly improved survival in patients with NSCLC, but at 5 years the absolute benefit was only about 5% [3]. Compared with chemotherapy alone, the addition of bevacizumab to adjuvant chemotherapy has failed to improve overall survival (OS) or disease-free survival (DFS) in patients with stage IB–IIIA disease [4]. Thus, the efficacy of adjuvant chemotherapy is not satisfactory.

EGFR mutations were identified in 40–60% of lung adenocarcinomas in East Asians, and exon19 and exon 21 accounted for 87% [5, 6]. Epidermal growth factor receptor tyrosine kinase inhibitors (EGFR-TKIs) have revolutionized the treatment of advanced NSCLC, leading to the investigation of adjuvant EGFR-TKI treatment for early-stage disease. The BR19 study demonstrated that gefitinib as an adjuvant treatment did not improve OS or progression-free survival compared with a placebo [7]. The RADIANT study failed to show that erlotinib as an adjuvant treatment prolonged DFS in EGFR-expressing NSCLC patients, but a benefit trend was observed in an EGFR-mutant subgroup [8]. The single-arm SELECT study demonstrated that adjuvant erlotinib improved the 2-year DFS rate (88%) in stage IA–IIIA patients with EGFR mutation compared to that of historic genotype-matched controls 76% [9].

The ADJUVANT and EVAN studies confirmed that in stage II–IIIA patients with EGFR mutation, adjuvant gefitinib or erlotinib significantly prolonged DFS compared with chemotherapy [10, 11]. However, the WJOG6410L study showed that adjuvant gefitinib did not significantly prolong DFS or OS in patients with completely resected stage II–III EGFR-mutated NSCLC [12]. In 2020, the ADAURA study showed the overwhelming efficacy of osimertinib for adjuvant treatment of patients with stage IB–IIIA NSCLC harboring EGFR mutation [13]. More recently, the EVIDENCE study showed encouraging results of adjuvant icotinib treatment of EGFR-mutant patients [14].

Several meta-analyses have been performed to assess the role of adjuvant EGFR-TKIs in resected patients [15, 16]; however, those studies did not include the ADAURA, WJOG6410L, or EVIDENCE studies. Moreover, the ADJUVANT and EVAN studies recently updated their OS data [17, 18]. Thus, we performed a meta-analysis of randomized controlled trials to evaluate the efficacy of EGFR-TKIs as adjuvant treatment for completely resected NSCLC.

## Methods

### Search strategy

We carried out this meta-analysis in accordance with the Preferred Reporting Items for Systematic Reviews and Meta-Analyses (PRISMA) statement. The protocol was registered in PROSPERO (ID: CRD42022316481).

**Fig. 1:**
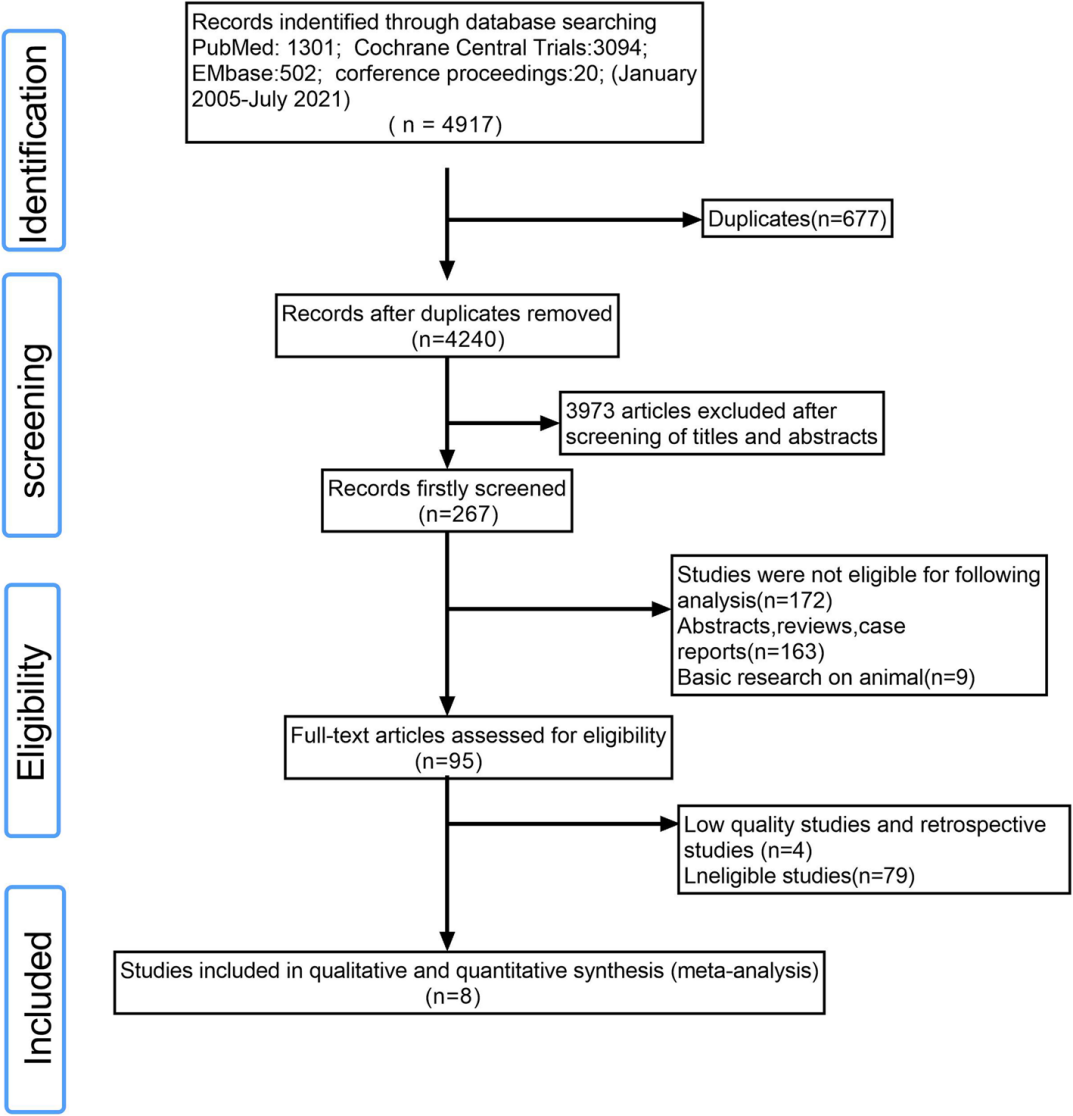
Flow chart of selection of studies to include or exclude

Two experienced investigators (Zhao and Wu) independently conducted a literature search and screening. They systematically searched the PubMed, EMBASE, Chinese Biomedical Literature Database, and Cochrane Library databases as the primary literature sources. The following conferences in the last 3 years were also searched manually: the *European Society for Medical Oncology*, the *American Society of Clinical Oncology* (ASCO), and the *World Conference on Lung Cancer*. The publishing deadline was July 1, 2021. Search keywords included non-small cell lung cancer, NSCLC, osimertinib, icotinib, gefitinib, erlotinib, afatinib, dacomitinib, and EGFR-TKI. The reference lists of studies and previous meta-analyses were also further investigated. Figure 1 presents the screening process.

### Eligibility criteria

The inclusion criteria were (1) phase II or III prospective randomized controlled clinical studies, (2) patients with histologically confirmed stage IB–IIIA NSCLC who had completed resection, and (3) patients who received EGFR-TKIs as adjuvant therapy. The exclusion criteria were (1) retrospective or non-randomized controlled clinical studies; (2) studies on the use of EGFR-TKIs as the first-line treatment for advanced lung cancer or the inclusion of immunotherapy in adjuvant therapy; (3) studies on wild-type EGFR (for EGFR-unselected patients, EGFR mutation data should be available); (4) case reports, reviews, and studies not written in English; and (5) articles with low quality or with small sample sizes (< 10 cases).

### Data extraction and quality assessment

The following data were extracted from included studies: authors, publication year, number of patients, patient characteristics, stage, EGFR status, treatments, DFS, OS, hazard ratio (HR), and 95% confidence interval (95% CI). The characteristics and outcomes of the included trials as well as the data extracted from the trials were entered into RevMan version 5.3 (Cochrane Collaboration, Oxford, UK). Two experienced investigators (Zhao and Wu) evaluated the quality of the studies independently using the Cochrane Risk of Bias tool. Sequence generation, allocation concealment, blinding, incomplete data, selective reporting, and other bias sources were assessed. Items were scored as having a low, high, or unclear risk of bias. Any discrepancies were determined by a third researcher (Yang).

### Statistical analyses

Statistical analyses were performed in Review Manager (Revman) Version 5.3. HRs were calculated for DFS and OS using the inverse variance method. *P* < 0.05 was considered statistically significant. All* P* values and 95% CIs were two-sided. Heterogeneity was assessed using the chi-squared test. If significant heterogeneity was high (*P* ≤ 0.05 or *I*^2^ > 50%), a random-effects model was used to reduce the impact of heterogeneity on the results; otherwise, a fixed-effect model was used. HRs were calculated as an effect measure for OS and DFS using the inverse variance method. Publication bias was tested using a funnel plot.

### Endpoints

The primary endpoint was DFS in EGFR-mutant patients, and other endpoints included DFS in subgroup analysis and OS.

## Results

### Eligible studies

**Fig. 2:**
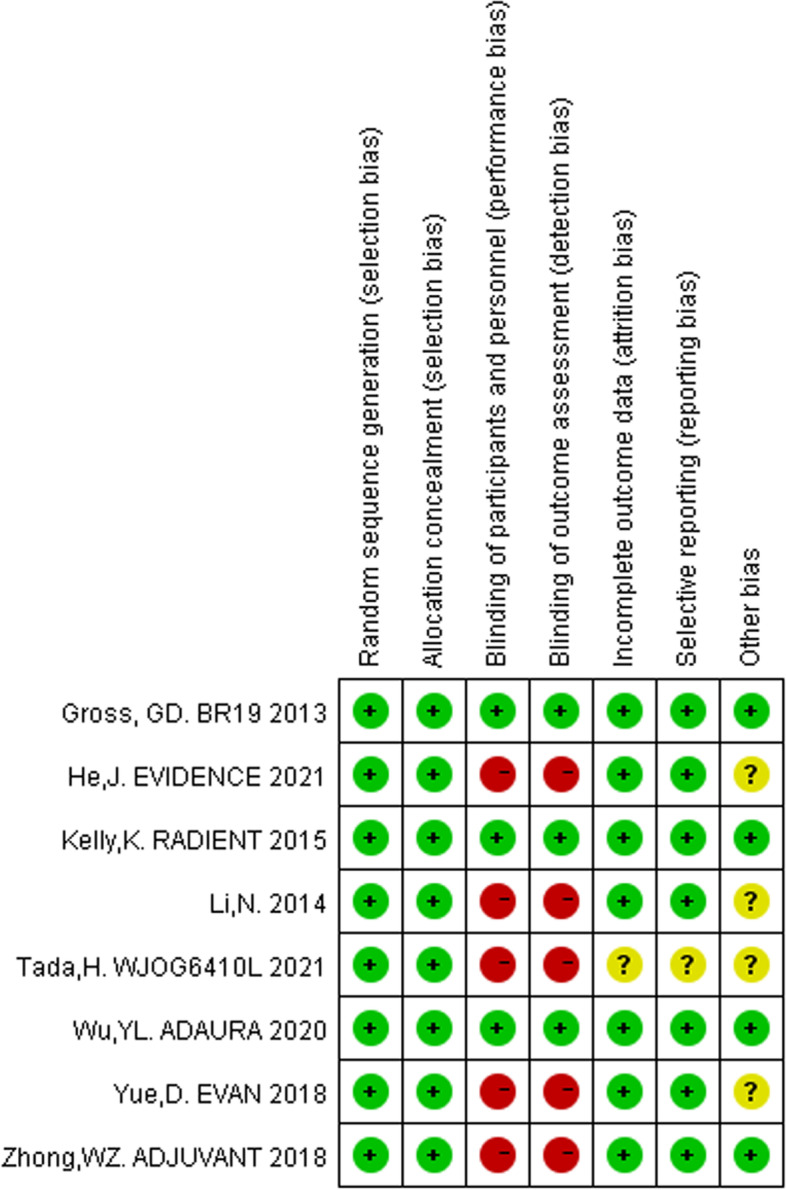
Risk of bias for each included study

Eight studies with a total of 3098 patients were included [7, 8, 10–14, 19]. Among these studies, six were phase III clinical trials and two were phase II clinical trials. Another study was excluded because it was of low quality, and the HRs were not available [20]. The characteristics of the studies included in the meta-analysis are shown in Table 1. We evaluated the quality of the studies using the Cochrane Risk of Bias tool, as shown in Fig. 2.

**Table 1:** Characteristics of the included studies

Author, Study	Year	Phase	Experimental	Control	Patients number	Races	Gender	Smoking status	Histology	Stage	EGFR status	Treatment duration	DFS (HR,95%CI)	OS (HR,95%CI)
Goss, G D BR19 [7]	2013	III	Gefitinib	Placebo	251 vs 252	White: 233 vs 235 Asian: 6 vs 3 other 12 vs 14	Male: 116 vs 116 female: 135%vs 136	Smoker: 224 vs 223 non-smoker: 27 vs 29	Adeno:150 vs 149 Non-adeno:101 vs 103	IB-IIIA	EGFR-M: 7 vs 8 wild type: 166 vs 178 Unknown: 78 vs 66	2 years	HR = 1.22 (0.93–1.61)	HR = 1.24 (0.94–1.64)
Li, N [19]	2014	II	Gefitinib + pemetrexed + carboplatin	Pemetrexed + carboplatin	30 vs 30	Asian: 30 vs 30	Male: 17 vs 18 female: 13 vs 12	Smoker: 13 vs 14 non-smoker: 17 vs 16	Adeno:28 vs 228 Non-adeno: 2 vs 2	IIIA	19-del: 11 vs 9 L858R: 19 vs 21	6 months	HR = 0.37 (0.16–0.85)	HR = 0.37 (0.12–1.11)
Kelly, K RADIANT [8]	2015	III	Erlotinib	Placebo	623 vs 350	White: 500 vs 279 Asian: 107 vs 60 other 16 vs 11	Male: 366 vs 209 female: 257 vs 141	Smoker: 494 vs 280 non-smoker: 129 vs 70	Adeno:367 vs 211 Non-adeno: 256 vs 139	IB-IIIA	EGFR-M: 102 vs 59 wild type:458 vs 245 Unknown: 29 vs 16	2 years	HR = 0.90 (0.74–1.10)	HR = 1.13 (0.88–1.45)
Zhong, W Z ADJUVANT [10, 17]	2018	III	Gefitinib	Vinorelbine + cisplatin	111 vs 111	Asian: 111 vs 111	Male: 44 vs 45 female: 65 vs 65	Smoker: 29 vs 26 non-smoker: 82 vs 85	Adeno:102 vs 105 Non-adeno: 9 vs 6	IIA-IIIA	19-del: 58 vs 57 L858R: 53 vs 53	2 years	HR = 0.60 (0.42–0.87)	HR = 0.92 (0.62–1.36)
Yue, D EVAN [11, 18]	2018	II	Erlotinib	Vinorelbine + cisplatin	51 vs 51	Asian: 51 vs 51	Male: 17 vs 20 female: 34 vs 31	Smoker: 13 vs 12 non-smoker: 38 vs 39	Adeno:46 vs 45 Non-adeno: 5 vs 6	IIIA	19-del: 30 vs 28 L858R: 21vs 23	2 years	HR = 0.27 (0.14–0.53)	HR = 0.17 (0.05–0.58)
Wu, Y L ADAURA [13]	2020	III	Osimertinib	Placebo	339 vs 343	Asian: 217 vs 220 non-Asian:122 123	Male: 108 vs 96 female: 231 vs 247	Smoker: 108 vs 86 non-smoker: 231 vs 257	Adeno:325 vs 332 Non-adeno: 14 vs 11	IB-IIIA	19-del: 186 vs 189 L858R: 153 vs 154	3 years	HR = 0.17 (0.11–0.26)	HR = 0.40 (0.09–1.83)
He, J EVIDENCE [14]	2021	III	Icotinib	Vinorelbine + cisplatin	151 vs 132	Asian: 151 vs 132	Male: 77 vs 55 female: 74 vs 77	Smoker: 49 vs 40 non-smoker: 102 vs 92	Adeno:143 vs 129 Non-adeno: 8 vs 3	IIA-IIIA	19-del: 80 vs 70 L858R: 71vs 62	2 years	HR = 0.36 (0.24–0.55)	HR = 0.91 (0.42–1.94)
Tada, H WJOG6410L [12]	2021	III	Gefitinib	Vinorelbine + cisplatin	116 vs 116	Asian: 116 vs 116	Male: 44 vs 45 female: 72 vs 71	Smoker: 48 vs 42 non-smoker: 68 vs 74	/	IIA-IIIA	19-del: 64 vs 59 L858R: 52 vs 56	2 years	HR = 0.92 (0.67–1.28)	HR = 1.03 (0.65–1.65)

### DFS

**Fig. 3:**
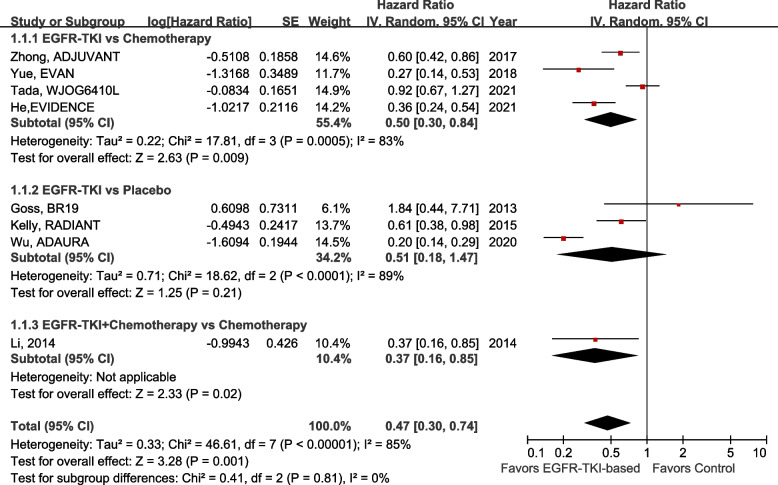
Forest plot of DFS in EGFR-TKI- vs. non-EGFR-TKI-treated patients

Data on median DFS and HRs were available in all eight trials. As shown in Fig. 3, in EGFR-mutant patients, the DFS of the adjuvant EGFR-TKI group was significantly superior to that of the control group, with a HR of 0.47 (95% CI 0.30–0.74; *P* = 0.001). Compared with chemotherapy, DFS was significantly improved in the EGFR-TKI subgroup (HR 0.50, 95% CI 0.30–0.84; *P* = 0.009), whereas DFS did not differ significantly between the placebo and EGFR-TKI subgroups (HR 0.68, 95% CI 0.21–2.17; *P* = 0.51). Only one study compared EGFR-TKI combined with chemotherapy to chemotherapy alone, showing significant improvement in DFS with a HR of 0.37 (95% CI 0.16–0.85; *P* = 0.02).

### Subgroup analysis of DFS

**Fig. 4:**
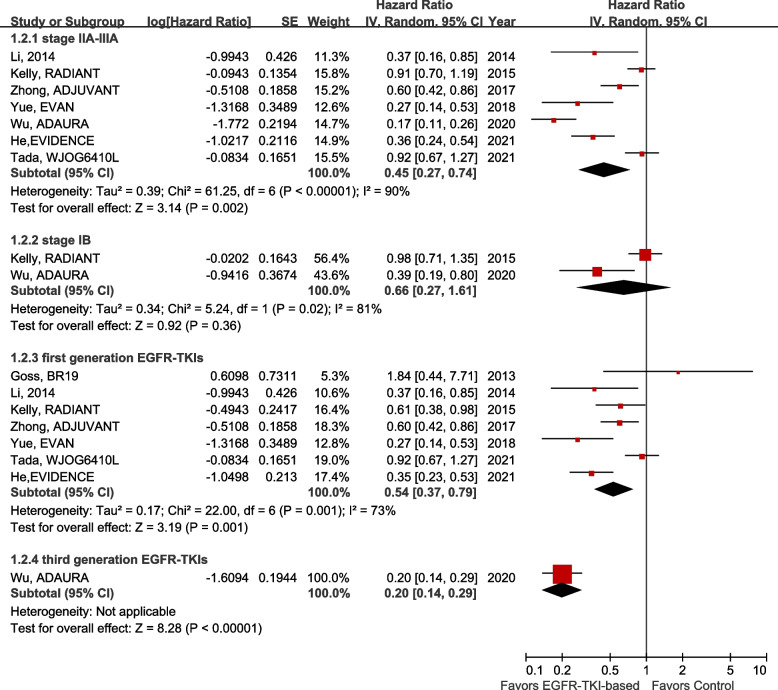
Forest plot of DFS in the subgroup analysis of EGFR-mutant patients

We performed a subgroup analysis of DFS. As shown in the forest plot (Fig. 4), in patients with stage IIA–IIIA NSCLC, DFS was significantly prolonged in the EGFR-TKI group compared with the control group (HR 0.45, 95% CI 0.27–0.74; *P* = 0.002). However, in stage IB patients, the difference in DFS between the EGFR-TKI and control groups was not significant (HR 0.66, 95% CI 0.27–1.61; *P* = 0.36). Seven studies used the first-generation EGFR-TKIs; the overall HR for DFS in this subgroup was 0.54 (95% CI 0.37–0.79; *P* = 0.001). Only the ADAURA study used the third-generation EGFR-TKI osimertinib, with a superior HR of 0.20 (95% CI 0.14–0.30; *P* < 0.001).

### OS

**Fig. 5:**
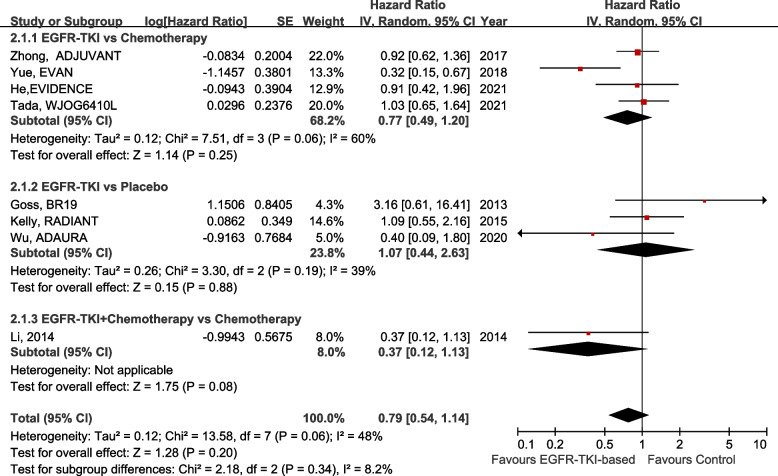
Forest plot of OS in EGFR-TKI- vs. non-EGFR-TKI-treated patients

The forest plot of OS implied that the difference between the adjuvant EGFR-TKI group and the control group was not statistically significant (HR 0.79, 95% CI 0.54–1.14, *P* = 0.20). No significant differences were found among all subgroups. In the subgroup of EGFR-TKI compared with chemotherapy, the HR was 0.77 (95% CI 0.49–1.20, *P* = 0.25). In the EGFR-TKI versus placebo subgroup, the overall HR was 1.07 (95% CI 0.44–2.63; *P* = 0.88) (Fig. 5).

## Discussion

In this study, we performed a meta-analysis to assess the efficacy of EGFR-TKIs as adjuvant treatment for completely resected NSCLC. The results reached the first endpoint. The DFS of the adjuvant EGFR-TKI group was significantly superior to that of the control group in EGFR-mutant patients, with a HR of 0.47 (*P* = 0.001), showing that adjuvant EGFR-TKIs decreased the risk of disease recurrence or death by 53% compared with the non-EGFR-TKI group. However, the benefit did not result in a significant improvement in OS (HR 0.79, *P* = 0.20), possibly due to the crossover effect of subsequent therapies. Moreover, the OS data of ADAURA and EVIDENCE were immature.

In patients with advanced NSCLC, the first-generation EGFR-TKIs have been shown to be superior to chemotherapy with respect to progression-free survival in untreated patients [21–23]. Adjuvant EGFR-TKI treatment is still being explored for the treatment of early-stage NSCLC, but it has been a challenging process. Initial studies, including BR19 and RADIANT, produced negative results because the patients enrolled in these two studies were unselected. Patients with wild-type EGFR did not benefit from EGFR-TKIs compared with chemotherapy [24]; thus, EGFR mutation status should be detected before the use of EGFR-TKIs. It is not recommended that patients with unknown EGFR mutation status use EGFR-TKIs in the adjuvant treatment setting.

Several questions remain about adjuvant EGFR-TKI treatment. First, it is unclear whether EGFR-TKIs should be used in stage IB EGFR-mutant patients after complete resection. In the ADJUVANT, EVAN, and EVIDENCE studies, all enrolled patients had stage II–IIIA disease with EGFR mutation, and the results showed improved DFS with EGFR-TKIs compared to chemotherapy. Only the WJOG6410L study showed that adjuvant gefitinib did not significantly prolong DFS or OS in patients with stage II–III NSCLC with EGFR mutation [12]. In this meta-analysis, the overall DFS of stage IIA–IIIA NSCLC was significantly prolonged in the EGFR-TKI group, with a HR of 0.45 (*P* = 0.002), showing that the risk of disease recurrence or death decreased by 55% in the adjuvant EGFR-TKI group compared with the non-EGFR-TKI-treated group. Two studies (ADAURA, RADIANT) enrolled stage IB patients with available data, and their overall DFS was not significantly different (HR 0.66; *P* = 0.36). Only the ADAURA study found that EGFR-mutant patients with stage IB could also benefit from osimertinib, with a HR of 0.39 (95% CI 0.18–0.76), though the degree of benefit was smaller than that in stage II (HR 0.17; 95% CI 0.08–0.31) and stage IIIA (HR 0.12; 95% CI 0.07–0.20). Therefore, EGFR-mutant patients with stage II–IIIA disease, especially those with lymph node metastases, can benefit from adjuvant EGFR-TKI treatment after resection. Stage IB patients may benefit from third-generation TKIs rather than first-generation TKIs.

Second, it is unknown whether first-generation or third-generation TKIs should be used as adjuvant treatment in EGFR-mutant patients post-surgery. The efficacy of the third-generation EGFR-TKI osimertinib has been shown to be superior to that of first-generation TKIs for the first-line treatment of EGFR-mutant advanced NSCLC [25, 26]. In this meta-analysis, six studies used first-generation EGFR-TKIs and only one study used the third-generation EGFR-TKIs; however, the HR for DFS in the third-generation subgroup was superior, providing the best HR to date (HR 0.20; *P* < 0.001). Moreover, adjuvant osimertinib reduces the risk of central nervous system recurrence by 82% in patients with resected EGFR-mutant NSCLC [13]. The overwhelming efficacy of osimertinib has made it the priority adjuvant treatment for EGFR-mutant patients. However, subsequent treatment should be considered if third-generation TKIs are used in the adjuvant setting; the final OS results of the ADAURA study will be relevant and should elucidate this issue.

Third, what role of adjuvant chemotherapy play in EGFR-mutant patients after complete resection? Chemotherapy improves survival rates by about 5% in patients with NSCLC, and adjuvant chemotherapy is the standard treatment in patients with completely resected stage II–IIIA disease [27]. In patients harboring EGFR mutation, three head-to-head studies (including ADJUVANT, EVAN, and EVIDENCE) demonstrated that EGFR-TKIs were superior to chemotherapy as adjuvant therapy. However, the WJOG6410L study presented at the 2021 ASCO meeting found that adjuvant gefitinib did not significantly prolong DFS or OS compared with chemotherapy. In this meta-analysis, the overall HR for EGFR-TKIs compared with chemotherapy was 0.50 (*P* = 0.009), while in the EGFR-TKI combined with chemotherapy versus the chemotherapy-alone subgroup, the difference in DFS was not significant (HR 0.38; *P* = 0.11). In the ADAURA study, the majority of patients with stage II–IIIA disease and approximately one-quarter of stage IB patients received adjuvant chemotherapy. The HRs for DFS were 0.16 (0.10–0.26) and 0.23 (0.13–0.40) in patients who did and did not receive adjuvant chemotherapy, respectively. The subsequent analysis of the ADAURA study showed adjuvant osimertinib as an effective treatment for patients with stage IB–IIIA EGFR mutation NSCLC after resection, with or without prior adjuvant chemotherapy [28]. Adjuvant chemotherapy appeared to have a limited effect on EGFR-mutant patients after resection. In a multicenter retrospective study, researchers found adjuvant EGFT-TKIs might be a beneficial choice compared with EGFR-TKIs plus chemotherapy in EGFR-mutant stage III-pN2 lung adenocarcinoma [29]. These results do not indicate that adjuvant chemotherapy should be abandoned. To date, adjuvant chemotherapy is one of the only treatments that has shown an OS benefit in resected NSCLC. Therefore, further prospective studies designed to understand the role of adjuvant chemotherapy in EGFR-mutant NSCLC patients are needed.

Fourth, the optimal exposure duration of adjuvant EGFR-TKI treatment is uncertain. For advanced patients, EGFR-TKIs are recommended until disease progression, but in the adjuvant setting, the situation is somewhat different. Patients can have long tumor-free survival after complete resection. Currently, most clinical trials are designed with 2 years of adjuvant drug treatment, but these studies have been empirical without definitive evidence. However, Lyu et al. found that 2 years of treatment with icotinib resulted in a significantly lower risk of recurrence compared to 1 year of treatment in EGFR-mutant patients with stage II–IIIA NSCLC after R0 resection [30]. The 3-year treatment duration in the ADAURA study led to great benefits. Whether a longer treatment duration might lead to improved survival remains unknown. A head-to-head clinical trial is needed for further analysis.

Finally, the benefit of DFS did not translate into improved OS, possibly due to the crossover effect of subsequent therapies. After disease recurrence, patients may receive many lines of treatment, which may contribute to improved OS. In the final OS analysis of the ADJUVANT study, the median OS times were 75.5 months and 62.8 months with gefitinib and chemotherapy, respectively (HR 0.92, *P* = 0 0.674) [17]. At the 2021 ASCO meeting, the OS of the EVAN study was updated; the median OS was 84.2 months with adjuvant erlotinib versus 61.1 months with adjuvant chemotherapy (HR 0.318; 95% CI 0.151–0.670) [18]. This was the first randomized study of adjuvant EGFR-TKI treatment to demonstrate a clinically meaningful improvement in OS versus chemotherapy alone in patients with stage IIIA EGFR-mutant NSCLC.

This meta-analysis had several limitations. First, the meta-analysis was not based on individual data, and some data were from subgroup analyses; moreover, the OS data of several studies were immature, which may have led to bias. Second, some studies compared EGFR-TKI treatment to chemotherapy while others compared it to a placebo, which made them not completely comparable. In addition, due to the limitations of the included studies, the optimal adjuvant treatment for patients with resected NSCLC harboring EGFR mutation is still unclear. The treatment model used in the ADAURA study was accepted by doctors, but longer follow-ups are needed. And new clinical trials that can result in changes in clinical practice merit further exploration.

In conclusion, EGFR-TKIs prolonged DFS but not OS in completely resected stage II–IIIA NSCLC patients harboring EGFR mutation. Longer follow-ups and new clinical trials that can result in changes in clinical practice are needed.

## Supplementary Information

**Additional file 1:** Search Strategy.

## Data Availability

Not applicable.
