# Supplementary material for: Epidermal growth factor receptor inhibitors as adjuvant treatment for patients with resected non-small cell lung cancer harboring EGFR mutation: a meta-analysis of randomized controlled clinical trials
Source: World J Surg Oncol. 2023 Feb 13;21:45. doi: 10.1186/s12957-023-02925-x (PMC9926719; doi:10.1186/s12957-023-02925-x)
Supplement: Supplementary file 1 — Additional file 1: Search Strategy. [file 12957_2023_2925_MOESM1_ESM.docx]

| Database | Search Strategy |
| --- | --- |
| PubMed | ((((Non-Small Cell Lung Cancer[MeSH Major Topic]) OR (((((((Non-Small Cell Lung Cancer[Title/Abstract]) OR (Non-Small Cell Lung Carcinoma[Title/Abstract])) OR (NSCLC[Title/Abstract])) OR (Non Small Cell Lung Carcinoma[Title/Abstract])) OR (Non-Small-Cell Lung Carcinoma[Title/Abstract])) OR (Non-small Cell Lung Cancer[Title/Abstract])) OR (Non-Small-Cell Lung Carcinomas[Title/Abstract])) OR (Non-Small-Cell Lung Carcinoma[Title/Abstract]))))) AND ((((((tyrosine kinase inhibitor) OR (TKI)) OR (epidermal growth factor receptor tyrosine kinases inhibitor)) OR (EGFR-TKI)) OR (EGFR mutation)) OR (("Chemotherapy, Adjuvant"[Mesh]) OR ((((Drug Therapy[Title/Abstract]) OR (Adjuvant[Title/Abstract])) OR (Adjuvant Chemotherapy[Title/Abstract])) OR (Adjuvant Drug Therapy[Title/Abstract])))) AND (1000/1/1:2021/7/1[pdat]) |
| Embase | ('epidermal growth factor receptor-tyrosine kinase inhibitor':ti,ab OR 'egfr-tki':ti,ab OR 'egfr':ab,ti OR 'adjuvant therapy':ab,ti OR 'adjuvant treatment':ab,ti) AND ('non small cell' AND ('lung'/exp OR lung) OR 'carcinoma, non small cell lung':ab,ti OR 'non-small-cell lung carcinoma':ab,ti OR 'lung carcinoma, non-small-cell':ab,ti OR 'non-small cell lung cancer':ab,ti) AND [1-1-0001]/sd NOT [1-7-2021]/sd |
| Cochrane library | ((MeSH descriptor: [Carcinoma, Non-Small-Cell Lung] explode all trees) OR (Non-Small Cell Lung Cancer):ti,ab,kw OR (NSCLC):ti,ab,kw OR (Non-Small Cell Lung Carcinoma):ti,ab,kw OR (Non Small Cell Lung Carcinoma):ti,ab,kw (Non-Small-Cell Lung Carcinoma):ti,ab,kw OR (Non-small Cell Lung Cancer):ti,ab,kw OR (Non-Small-Cell Lung Carcinomas):ti,ab,kw OR (Non-Small-Cell Lung Carcinoma):ti,ab,kw) AND ((epidermal growth factor receptor-tyrosine kinase inhibitor):ti,ab,kw OR (egfr-tki):ti,ab,kw OR (EGFR):ti,ab,kw OR (adjuvant therapy):ti,ab,kw OR (adjuvant treatment):ti,ab,kw) with Cochrane Library publication date from Jan 0001 to Jul 2021 |
| Chinese Biomedical Literature Database | (("EGFR-TKI"[全部字段] OR "表皮生长因子受体酪氨酸激酶抑制剂"[全部字段] OR "辅助治疗"[全部字段] OR "辅助靶向治疗"[全部字段] OR "达克替尼"[全部字段] OR "阿法替尼"[全部字段] OR "埃克替尼"[全部字段] OR ("厄洛替尼"[全部字段] OR "盐酸厄洛替尼"[全部字段] OR "11C-埃罗替尼"[全部字段] OR "774 CP 358"[全部字段] OR "CP 358774"[全部字段] OR "774 CP-358"[全部字段] OR "CP-358774"[全部字段] OR "盐酸埃罗替尼"[全部字段] OR "7-双(2-甲氧基乙氧基)喹唑啉-4-胺N-(3-乙炔苯基)-6"[全部字段] OR "OSI-774"[全部字段] OR "它赛瓦"[全部字段] OR "盐酸厄洛替尼"[主题词]) OR "奥希替尼"[全部字段] OR "吉非替尼"[全部字段]) AND (("肺癌"[全部字段] OR "肺肿瘤"[全部字段] OR "肺部癌症"[全部字段] OR "肺部肿瘤"[全部字段] OR "肺肿瘤"[主题词]) OR "癌, 非小细胞肺"[不加权:扩展])) AND 2012-2022[日期] |
